# Fat harvesting protocol for enhanced stem cell viability – pilot study

**DOI:** 10.1016/j.clinsp.2026.100945

**Published:** 2026-04-24

**Authors:** Ruan Pimenta, Vanessa Ribeiro Guimarães, Samirah Abreu Gomes, Silvana Cereijido Altran, Emily Caroline da Fonseca Sampaio, Elaine Rufo Tavares, Rolf Gemperli, Cristina Pires Camargo

**Affiliations:** a Laboratory of Medical Research, Department of Urology, Faculdade de Medicina da Universidade de São Paulo, São Paulo, SP, Brazil; b Cell Therapy and Tissue Engineering Unit (UTC-ET), Hospital das Clínicas da Faculdade de Medicina da Universidade de São Paulo, São Paulo, SP, Brazil; c Laboratório de Nefrologia Celular, Genética e Molecular, Departamento de Clínica Médica, Faculdade de Medicina da Universidade de São Paulo, São Paulo, SP, Brazil; d Microsurgery and Plastic Surgery Laboratory (LIM-04), Faculdade de Medicina da Universidade de São Paulo, São Paulo, SP, Brazil; e Heart Institute, Hospital das Clínicas da Faculdade de Medicina da Universidade de São Paulo, São Paulo, SP, Brazil

**Keywords:** Fat tissue, Adipose tissue, Adipocyte, Fat graft, Stem Cells

## Abstract

**Objective:**

Adipose tissue is used in several specialties to obtain Adipose-derived Stem Cells (ASC) and fat derivatives. Several studies analyze protocols for harvesting fat tissues. However, these studies showed inconclusive data regarding cell viability and function. Thus, the objective of this study was to analyze protocols regarding cell viability, identify the best method, and test cell differentiation and proliferation when using the chosen methods.

**Methods:**

This study harvested lipoaspirate products from three healthy women undergoing aesthetic procedures. The first analysis corresponds to the viability of fat tissue liposuction products for the washing process. The second analyzes the effect of anesthetic drugs, and the third analyzes the cytotoxicity of the fraction process. After testing all these steps, the authors prepared the fat tissue using the best method chosen after the first analysis to test cell differentiation (adipogenesis, osteogenesis, and chondrogenesis) and proliferation.

**Results:**

The analysis showed that saline solution 0.9% was the washing solution that induced less cell death. Regarding anesthetic and vasoconstrictor drugs, lidocaine, ropivacaine, and epinephrine caused similar levels of cell death, but sodium bicarbonate increased cell proliferation. The number of passages through a 2.4 mm Luer-to-Luer transfer device did not influence cell viability. The proliferation and differentiation of ASC in adipocytes, osteoblasts, and chondrocytes were preserved using saline solution 0.9% and 20 × passages for fat fractioning.

**Conclusions:**

The findings of this study provide preliminary in vitro evidence that may improve future investigations on the fat harvesting process.

## Highlights

• Multiple methods exist to extract Adipose-Derived Stem Cells (ASCs) and fat derivatives for medical use.
• Saline solution 0.9% was the washing solution that showed the lowest rate of cell death; lidocaine, ropivacaine, and epinephrine caused similar levels of cell death, and passing fat through a 2.4 mm Luer-to-Luer connector up to 20 times did not affect cell viability.
• ASC proliferation and differentiation capability into adipocytes, osteocytes, and chondrocytes remained intact with saline and 20 × passages.
• These findings may enhance fat graft viability.

## Introduction

Fat grafts and fat fractions (stromal vascular fraction, stem cell culture) are routinely used as treatment strategies in plastic surgery.^1^^,^^2^ According to literature data, 85% of plastic surgeons in the USA use fat grafts associated with other surgical procedures.^3^

The routine use of fat grafting is due to several advantages, such as the noninvasive collection of adipose tissue, large available volume, and high yield for obtaining stem cells (3%‒5% of adult mesenchymal Stem Cells [SC], 2:500-times yield greater than bone marrow-derived CT).^1^ However, the retention rate of the grafted volume over time is very variable. After one year of follow-up, the fat retention rate can vary from 20%‒50%.^4^^,^^5^

This variability of results (retention rate) presents several challenges. The retention rate is based on neoangiogenesis; due to the significant volume of fat graft, the fat integration (by angiogenesis) does not reach the center of the graft, causing fat necrosis and a decrease in the retention rate. Another issue that interferes with the fat graft retention rate is the fat harvesting process. During surgical procedures, several substances are infiltrated into the fatty tissue, such as anesthetics and vasoconstrictors, which can interfere with the viability of fat cells and, consequently, adipocyte-derived stem cells. Other factors that can still reduce cell viability include the fat processing technique (washing, fractionation).^3^^,^^6^^,^^7^

These variables can increase the apoptosis rate of adipose cells and Adipose-Derived Stem Cells (ADSC) contained in the tissue, consequently leading to low-fat graft retention.^6^^,^^7^

Decreased tissue viability can negatively impact the viability of stem cells contained in adipose tissue. The literature shows that stem cells can benefit from the fat retention rate due to their proliferative potential and potential paracrine effect.^1^^,^^8^ Therefore, there is a need to analyze the fat harvesting and processing protocols to increase cell viability and thereby increase the retention rate of fat grafts.^6^, ^7^, ^8^, ^9^, ^10^, ^11^

By efficiently processing adipose tissue, it is possible to produce fat fractions with better quality for grafting,^11^ vascular stromal fractions,^9^ enriched stem cell culture medium,^11^ and exosomes.^10^^,^^12^

Given this scenario, several techniques for obtaining fat derivatives can be analyzed. From handling fat collection (surgery) to using enzymatic and mechanical methods.^13^^,^^14^ Several authors analyzed different protocols for anesthesia, washing, and fractionation. However, these studies present inconclusive data regarding cell viability after adipose tissue harvesting, which may compromise cell analysis. Therefore, this study will analyze several fat processing protocols regarding each applied method's cell viability and adipose tissue cell proliferation potential.

## Methods

This experimental study validates a protocol for the adipose tissue processing for fat graft purposes. This study was a collaborative work of the Laboratory of Microsurgery and Plastic Surgery (LIM-4) of the *Hospital das Clínicas da Faculdade de Medicina da Universidade de São Paulo* (HCFMUSP) and the Cell Therapy and Tissue Engineering Unit (UTC-ET) of the HCFMUSP. This study followed the national guidelines for good practices and the use of laboratory animals, as established by the National Council for the Control of Animal Experimentation (CONCEA), and is in accordance with the ARRIVE guidelines. The study was approved by the Ethics Committee of HCFMUSP (CAPPesq-CAAE-68,296,023.4.0000.0068). All the participants signed the informed consent form. All participants were recruited from the Plastic Surgery Department, HCFMUSP.

### Inclusion criteria

The liposuction product was donated by three healthy female patients with the same basic demographic characteristics (patients without metabolic systemic disease). The age of the patients ranged from 44 to 47-years, and the mean BMI was 25.6. The patients underwent abdominoplasty and liposuction for aesthetic reasons. The donor site was the abdominal region.

### Exclusion criteria

Active systemic or local infection or uncontrolled systemic disease. Allergy to any component of the liposuction procedure.

### Surgical procedure (liposuction)

Three healthy female participants underwent the liposuction procedure for aesthetic reasons. The participants were put under general anesthesia in a decubitus dorsal position and submitted to antisepsis with chlorohexidine 0.3% (Rioquímica, São José do Rio Preto, Brazil). With a scalpel number 15, the authors made a 5 mm incision in the umbilicus and the superior iliac crest. A 4 mm multiperforated cannula was then inserted into the adipose tissue. During the liposuction procedure, the authors did not inject any substance into the subcutaneous compartment.

Using Coleman's technique, the research team collected ten 10 mL syringes (Luer-lock, BD, São Paulo, Brazil) of adipose tissue from the abdominal region, totaling 100 mL per participant.^15^

The samples of the three participants were mixed, producing a single sample with which all experiments were performed. The fat samples were decanted for 15 min. Then the authors divided the fat tissue into 10 mL aliquots to undergo the following steps independently:

• Analysis 1: Influence of the washing process of liposuction products using saline solution 0.9% or Ringer lactate on the viability and functionality of the adipose cells.
• Analysis 2: Influence of the use of anesthetics and vasoconstrictors on the viability and functionality of the adipose cells.
• Analysis 3: Influence of the number of passages during the fractioning process of adipose tissue on the viability and functionality of the adipose cells.
• Analysis 4: After defining the best washing solution, the anesthetics, and the fractioning procedure, the authors applied these techniques to the liposuction product and cultivated it to analyze colony unit formation and differentiation in adipocytes, cartilaginous cells and osteoblasts.

### Analysis 1 ‒ influence of the washing solutions on the viability of adipose tissue cells

Following the fat liposuction surgical procedure, the authors utilized gravity separation to isolate the fat components. All the syringes were placed in an upright position for 30 min at room temperature. The liquid phase at the bottom of each syringe was discarded. The liposuction product (fragmented with tweezers and scissors) and the stromal fraction (obtained by centrifugation at 1000 rpm for 3 min) were divided into 2 *g*roups:

• Saline solution 0.9% (Cristália, São Paulo, Brazil) group: 3 syringes were washed three times with saline solution.
• Ringer lactate (Isofarma, Fortaleza, Brazil) group: 3 syringes were washed three times with Ringer lactate solution.

After the homogenization and incubation of the samples with the different washing solutions, the authors performed the MTT analysis.

### Analysis 2 ‒ influence of anesthetics and vasoconstrictor use on the viability of adipose tissue cells

After harvesting the fat, the authors separated 5 mL of fat into aliquots and manually fragments in 1 mm by using tweezers and scissors, and homogenized each aliquot with lidocaine (Cristália, São Paulo, Brazil), ropivacaine (Cristália, São Paulo, Brazil), epinephrine (Blau, São Paulo, Brazil), or sodium bicarbonate (Samtec, Ribeirão Preto, Brazil), as follows:

• Lidocaine group: added 2% lidocaine (0.007 mg) for 1h.^16^^,^^17^
• Ropivacaine group: added ropivacaine 2% (0.07 mg) for 1h.^16^^,^^17^
• Epinephrine group: added epinephrine 1:1000 (0.01 mg) for 1h.^16^^,^^17^
• Sodium bicarbonate group: added sodium bicarbonate (0.04 mg, 8.4%) for 1h.^16^^,^^17^
• Control group: no addition of any substances, sample rested in the same conditions as the other aliquots for 1 h.

After 1 hour, the authors used the homogenized samples to perform the MTT analysis.

### Analysis 3 ‒ influence of the number of passages during fat tissue fractionation on the viability of adipose tissue cells

After harvesting the fat tissue, the authors separated ten syringes of 10 mL and divided them into four groups with 5 mL per vial triplicate.

• Control.
• Fat fractioning with a 2.4 mm Luer-to-Luer transfer (Tulip Medical, San Diego, USA) for 10-times.
• Fat fractioning with a 2.4 mm Luer-to-Luer transfer for 20 times.
• •Fat fractioning with a 2.4 mm Luer-to-Luer transfer for 30 times.

After the fractioning process, the authors proceeded with the MTT analysis.

### MTT analysis

MTT (3-(4,5-dimethylthiazol-2-yl)-2,5-diphenyl tetrazolium bromide) (Sigma-Aldrich, Burlington, USA) was solubilized in PBS at 5 mg/mL and filtered for sterilization and debris removal. The MTT solution was added to a 96-well plate.

Then, the homogenized samples of all three analyses were added to the plate. The authors performed the analysis of the washing solutions as 4 experiments in triplicate for each sample, and the analysis of the anesthetics and the number of passages of the fat fractioning process as three experiments in triplicate. After adding the homogenized fat, the plate was incubated at 37 °C for four hours. Acid-isopropanol (100/∼1 of 0.04 N HCI in isopropanol) was added to all wells and mixed thoroughly to dissolve the dark blue crystals. These mixtures rested at room temperature to ensure that all crystals were dissolved.

After this process, the plates were read within one hour of adding the isopropanol in a SpectraMax 340PC384 Microplate Reader (Molecular Devices, San Jose, USA), using a 560 nm filter.^18^

### Adipose-derived stem cell culture

Based on the analysis described above, the authors identified the optimal methods for washing, the appropriate use of anesthetics, and the ideal number of passages in the fractioning process. The authors then applied these procedures to the fat, using a 10 mL sample for culture and differentiation.

For enzymatic digestion, the material was transferred to a conical tube, containing 4 mL of trypsin/0.25% EDTA, placed in a water bath at 37 °C, under agitation every 5-minutes for 20-minutes. Trypsin inactivation was performed with the addition of 10 mL of DMEM culture medium (Dulbecco’s Modified Eagle’s Medium, Gibco, New York, USA), supplemented with 10% Fetal Bovine Serum (FBS) and 1% penicillin/streptomycin antibiotics.

The digested material was centrifuged at 1800 rpm for 5-minutes at 20 °C to obtain the cell pellet. The supernatant was carefully removed with a Pasteur pipette and washed with 10 mL of DMEM.

Subsequently, a new centrifugation was performed, the supernatant was discarded, and the pellet was resuspended in 2 mL of DMEM, where 10 µL were used to evaluate cell viability. The cells were placed in a Neubauer chamber with 0.4% Trypan blue dye and counted. The volume of the cell suspension was adjusted to 5 mL and placed in a 25 cm^2^ culture bottle, placed in an incubator, under a humid atmosphere containing 5% CO_2_ at 37 °C.

Cells were replicated whenever the confluence was greater than or equal to 80%. To detach cells adhered to the culture bottle, the enzymatic dissociation method was used, with trypsin/EDTA 0.25% for 2 min at 37 °C. The cells were expanded until the fourth passage. Next, the cells were subjected to enzymatic digestion with trypsin/EDTA 0.25% for 2-minutes at 37 °C. DMEM supplemented with 10% FBS was added to inactivate trypsin, and the cell suspension was centrifuged at 1.800 rpm for 5-minutes. The cells were transferred to a Neubauer chamber for counting and subsequent calculation to reach a concentration of 1 × 10^6^ cells/mL.

### Adipose-derived stem cell differentiation

For the differentiation assay, 1500 human ADSC cells were seeded in 6-well plates. After 4-days of culture for cell adhesion, the differentiation process was initiated. The Adipogenesis Differentiation Kit, Chondrogenesis Differentiation Kit and Osteogenesis Differentiation Kit (StemPro™, Gibco, New York, USA) were used. The kits are composed of basal differentiation medium (90%) and respective differentiation supplements (10%), and are added with antibiotic/antimycotic (Gibco, New York, USA) at a concentration of 5 µg/mL.

The plates were incubated at 37 °C and 5% CO_2_ for 14-days for adipogenic and chondrogenic differentiation and 21-days for osteogenic differentiation, changing the respective media every 3-days. For the controls, the authors used DMEM High Glucose (Gibco, New York, USA).

After the differentiation assay period, cells were stained to confirm the differentiation efficiency, and a specific dye was used for each lineage. For adipogenic differentiation, oil red O (Sigma-Aldrich, Burlington, USA) was used; for chondrogenic differentiation, alcian blue (Inlab, São Paulo, Brazil) and for osteogenic differentiation, alizarin red (Sigma-Aldrich, Burlington, USA) was used. The plates were then photographed using the PrimeCam 12 ProS equipment (Prime LifeScience, Boca Raton, USA).

### Proliferation assay

After confirming the differentiation, a proliferation experiment was performed to evaluate the ability of isolated cells to proliferate and form colonies. For this, the cells were plated at low density (300 cells/well) in 6-well plates and incubated at 37 °C and 5% CO_2_, and colony growth was observed for a period of 7- to 10-days. After this period, the cells were fixed with a 3:1 solution of methanol: acetic acid for 5-minutes.

Then, the authors performed staining to confirm the presence or absence of colonies. The cells were washed with PBS, then 1 mL of crystal violet dye (1%) was added, and the cells were incubated for 30-minutes. Then, the plates were washed to remove excess dye and the presence or absence of colonies was noted. The cells were also stained again with their respective dyes at this time to confirm whether they maintained differentiation. The plates were photographed using an inverted field microscope (Nikon, Tokyo, Japan) integrated with a PrimeCam Intervision 12 PRO camera. (Prime LifeScience, Boca Raton, USA).

### Statistical analysis

Due to the nature and distribution of the variables, data were presented as median and interquartile interval and percentage.

The cell viability assays were performed as 3 experiments in triplicate for the anesthetics and fat fractioning analysis, and 4 experiments in triplicate for the washing solutions analysis. In the washing protocol analysis, the authors used the Wilcoxon test to compare the saline solution and lactate ringer. In the following analysis, the authors used the Kruskal-Wallis test. If the outcome was significant, the authors used Dunn's test analysis. The authors considered an alpha value of 5% and the power of the study 80%. Statistical software STATA version 14 (Stata Corp, College Station, USA) was used for calculation.

## Results

### Analysis of washing solutions, anesthetics, and number of passages during tissue fractionation on adipose tissue cells viability

**Fig. 1:**
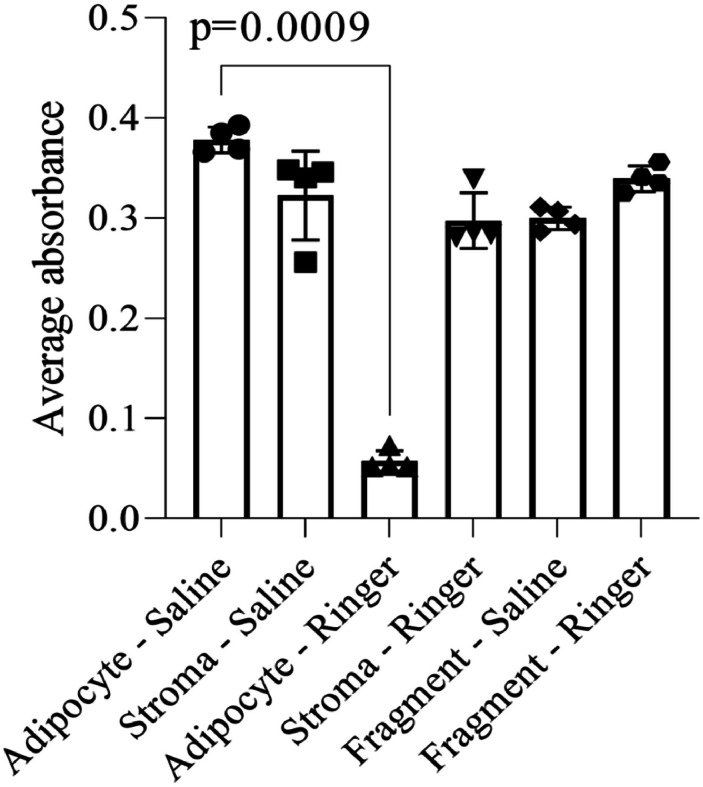
Cell viability assessment of adipose tissue samples subjected to various washing solutions. Samples include fragmented adipose tissue (liposuction product) and stromal fraction (obtained by centrifugation at 1000 rpm for 3 min). Viability was measured using the MTT assay, with absorbance read at 560 nm. Each data point represents the mean of triplicate measurements from a single experiment. Bar graphs display the mean ± standard deviation from four independent experiments.

The comparison between the washing solutions showed that saline solution caused less cell death compared to lactated Ringer's solution (p = 0.0009), which exhibited the highest rate of apoptosis (Fig. 1).

**Fig. 2:**
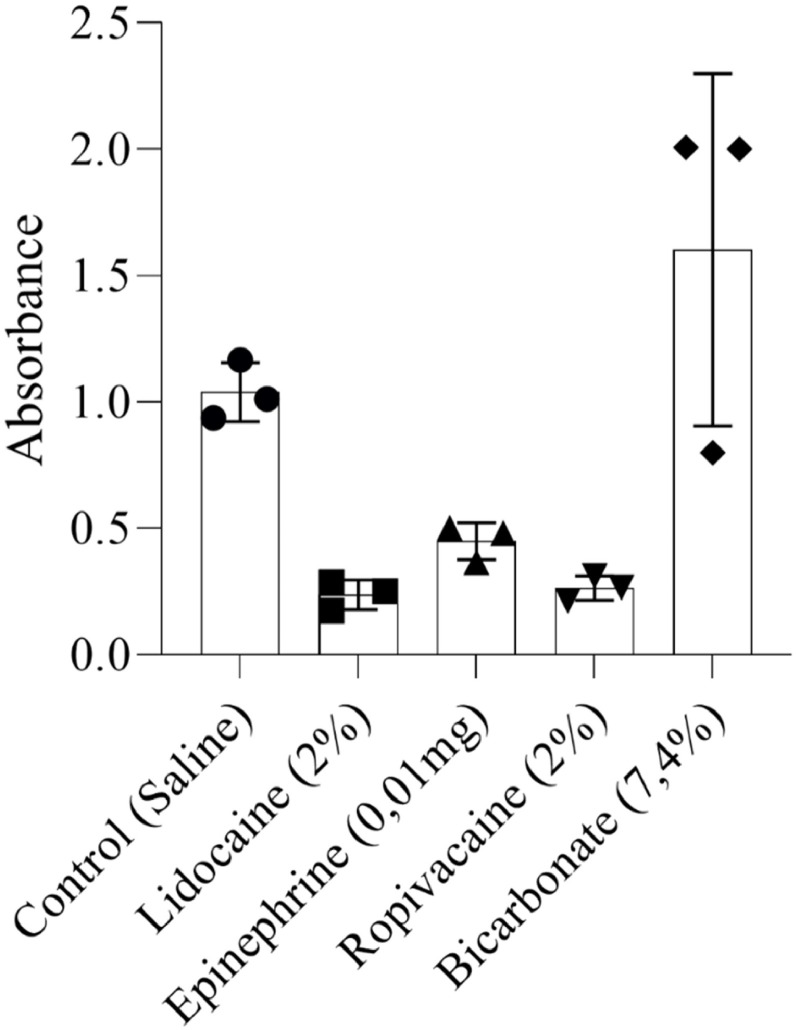
Cell viability analysis of fragmented adipose tissue (liposuction product) homogenized and exposed for 1 h to lidocaine, epinephrine, ropivacaine or sodium bicarbonate compared to saline solution 0.9% (Control). Viability was measured using the MTT assay, with absorbance read at 560 nm. Each data point represents the mean of triplicate measurements from a single experiment. Bar graphs display the mean ± standard deviation from three independent experiments.

Regarding the anesthetics and vasoconstrictor substances, only sodium bicarbonate did not promote apoptosis in the adipose tissue cells. Lidocaine, epinephrine and ropivacaine showed similar rates of cell death (Fig. 2).

**Fig. 3:**
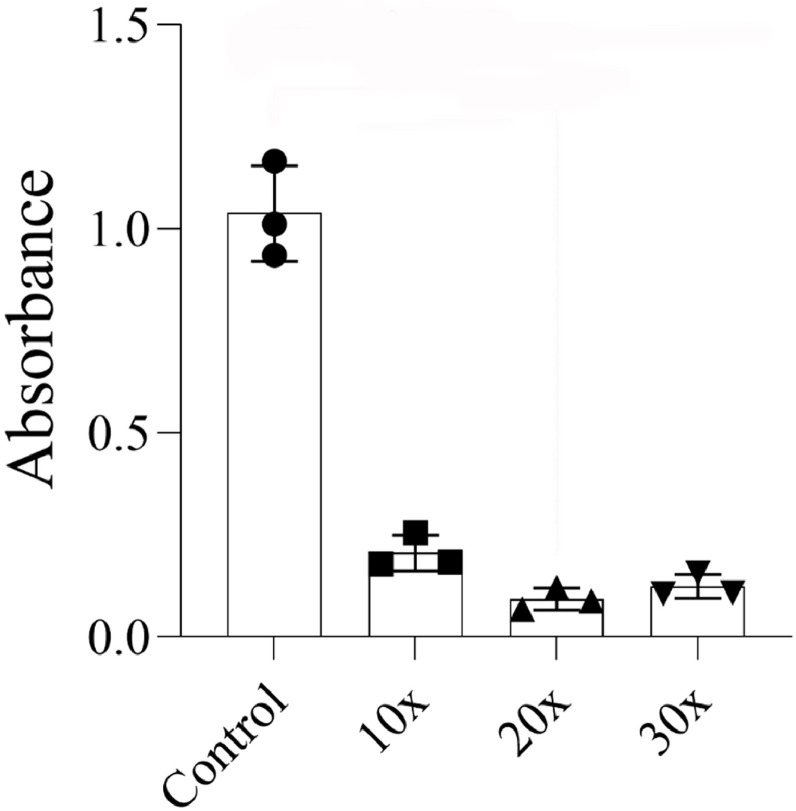
Cell viability analysis of the influence of the number of passages for fat fractioning with a 2.4 mm Luer-to-Luer transfer in adipose tissue viability. Viability was measured using the MTT assay, with absorbance read at 560 nm. Each data point represents the mean of triplicate measurements from a single experiment. Bar graphs display the mean ± standard deviation from three independent experiments.

The number of viable adipose tissue cells following the fractioning process decreased across all analyzed passages compared to the Control group, although the changes were not statistically significant (Fig. 3).

### Differentiation and proliferation of adipose-derived stem cells

**Fig. 4:**
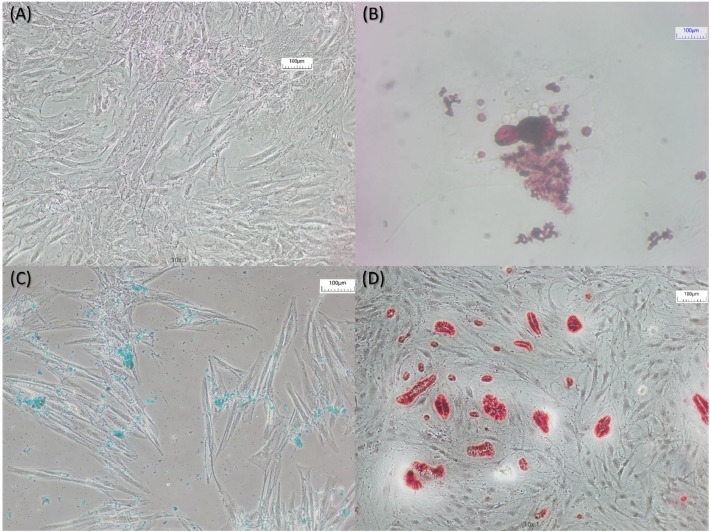
Photomicrographs of the differentiation assay showing (A) Control, (B) Adipocyte differentiation, (C) Chondrocyte differentiation, and (D) Osteoblast differentiation. The plates were incubated for 14-days for adipogenic and chondrogenesis and for 21-days for osteogenesis. The cells were then stained with (B) oil red O, (C) alcian blue, and (D) alizarin blue to confirm the differentiation. Magnification: 100 × . Scale bar: 100 µm.

The differentiation assay of the adipose-derived stem cells performed using saline solution 0.9% as washing solution and after 20 passages for fat fractioning showed that the adipose-derived stem cells successfully differentiated into adipocytes, chondrocytes, and osteoblasts (Fig. 4).

**Fig. 5:**
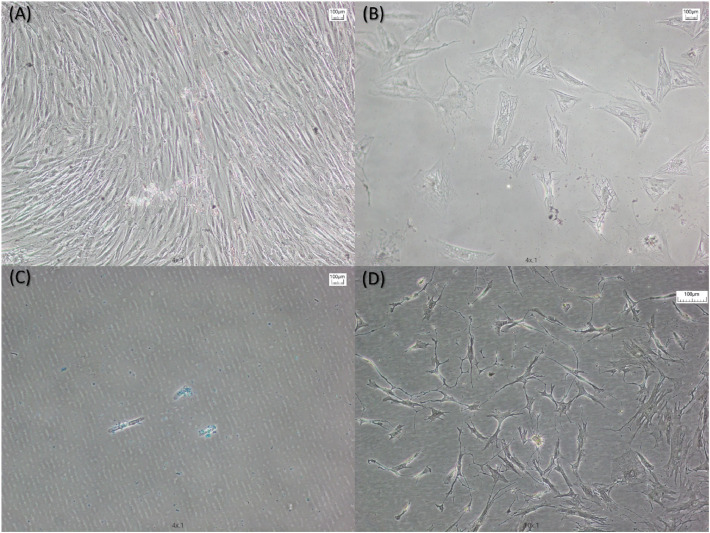
Photomicrographs of the proliferation assay. After confirming the differentiation of adipose-derived stem cells, the cells were cultivated for 7‒10 days and stained with (B) oil red O, (C) alcian blue, and (D) alizarin blue to verify if they maintained the differentiation. Magnification: 100 × . Scale bar: 100 µm.

The proliferation assay showed the presence of colonies of adipocytes, chondrocytes and osteoblasts derived from adipose-derived stem cells and the colonies maintained the differentiation at the end of the proliferation period (Fig. 5).

## Discussion

Effective harvesting and processing of fat tissue are crucial for optimal fat graft retention.^19^

Coleman established a reliable method for harvesting fat tissue.^15^^,^^20^ Following harvesting, it's essential to process the aspirated tissue to remove blood, fibrous tissue, and endothelial cells from fat. Various authors have analyzed different protocols for washing and fractionation procedures.^21^ Some employ centrifugation to separate these elements.^7^^,^^17^ However, several studies have suggested that the speed of centrifugation may influence the risk of apoptosis.^22^^,^^23^

Considering these controversies, the group conducted an analysis of the impact of anesthetics and vasoconstrictors used during harvesting, the processes involved in washing blood and fibrous elements from fat tissue, and the fractionation procedures. These findings are highly significant in the field, as they provide valuable insights into improving fat tissue processing techniques.

Regarding the fat tissue washing process, the present study showed that 0.9% saline solution caused less apoptosis than Ringer’s lactate solution. Interestingly, Smith et al.^20^ proposed a protocol using a Ringer’s lactate solution for fat tissue preparation for grafting. The study showed no difference in the apoptosis rate using saline solution or Ringer’s lactate solution. The authors exposed the tissue using the same methodology and observed that Ringer’s lactate-treated adipocytes showed a higher apoptosis rate.

Then, the authors analyzed the effect of different anesthetics and substances used in clinical practice. Keck et al.^16^ analyzed the effect of anesthetics (lidocaine, bupivacaine, epinephrine) on adipocyte cell cultures. Their study showed that all substances, except bupivacaine, reduced cell viability. These findings may overestimate the deleterious effect of anesthetics on stem cells due to the isolated condition of the cells.

In practical use, anesthetic solutions are injected into the tissue rather than directly into the ADSC. In this context, other cellular and structural elements may offer protection against anesthetic substances.

To test this hypothesis, the authors analyzed the adipose tissue after anesthetic infiltration (lidocaine, bupivacaine, and epinephrine) for proliferation and differentiation analysis. The findings showed that the anesthetics and vasoconstrictor substances worsened adipose tissue cells' viability. In other hand, some authors add sodium bicarbonate to increase local pH to improve the microenvironment and probably decrease cell apoptosis.^24^ In this sense, the authors tested the addition of sodium bicarbonate in the fat tissue. These findings showed less cell apoptosis in the sodium bicarbonate samples. Therefore, the authors hypothesized that sodium bicarbonate was not harmful to the cells. Similarly, Bugajska-Liedtke^25^ showed that the use of bicarbonate-buffered solution improved the survival of the ASC in 53% compared to the standard tumescent solution, composed of Ringer’s lactate, lidocaine, and epinephrine. The pH neutralization of the tumescent solution protected the ASC population.

Regarding the fraction procedure, it is clinically used to increase the surface contact of fat tissue, potentially enhancing cell nurture (imbibition) and ultimately improving the neoangiogenesis effect. In the present study, the authors analyzed different passages using a 2.4 mm tissue transfer. The diameter of this transfer equipment fragments the fat tissue samples and produces nanofat, which is rich in stem cells and used in tissue repair and regeneration. Despite the different number of transfers, there was no difference in the apoptosis rate.

Therefore, the present study demonstrated that, under these experimental and analytical conditions, the washing solution that causes the least apoptosis is 0.9% saline solution. Regarding anesthetics and vasoconstrictors, as all are detrimental to the cells at some level, an option is to use sodium bicarbonate to neutralize the acidity of the anesthetics, increasing the viability of adipocytes. As for the fractionation procedure with a 2.4 mm device, the number of passages does not influence cell death, allowing for the most feasible option to be chosen by the professional in charge. Finally, the processes that the adipose-derived stem cells were subjected to did not interfere negatively with the adipose-derived stem cell proliferation and differentiation.

## Limitations

This study had some limitations. The authors analyzed the fat derivative protocols in vitro. The next step will be to implant the differently processed fat tissue product in an animal model to test the viability and function.

## Conclusions

The findings of this study provide preliminary in vitro evidence that may improve future investigations on the fat harvesting process. However, these results are limited to cell culture experiments, and further animal studies are required to determine whether such effects could influence graft viability, retention, or clinical outcomes.

## Authors’ contributions

CPC: Conceptualization; Supervision; Writing.

RP: Investigation; Data curation.

VRG: Investigation; Data curation.

SAG: Methodology; Formal analysis.

SCA: Supervision; Methodology.

ECFS: Investigation; Data curation.

ERT: Writing review; Validation.

RG: Project administration; Writing.

## Funding

This study had no funding.

## Data availability statement

The data used and/or generated in this study are available from the corresponding author upon reasonable request.

## Conflicts of interest

The authors declare no conflicts of interest.

## References

[1] Samsonraj R.M., Raghunath M., Nurcombe V., Hui J.H., van Wijnen A.J., Cool S.M. (2017). Concise review: multifaceted characterization of Human mesenchymal stem cells for use in regenerative medicine. Stem Cells Transl Med.

[2] Mushahary D., Spittler A., Kasper C., Weber V., Charwat V. (2018). Isolation, cultivation, and characterization of human mesenchymal stem cells. Cytometry A.

[3] Strong A.L., Cederna P.S., Rubin J.P., Coleman S.R., Levi B. (2015). The current State of fat grafting: a review of harvesting, processing, and injection techniques. Plast Reconstr Surg.

[4] Trotzier C., Sequeira I., Auxenfans C., Mojallal A.A. (2023). Fat graft retention: adipose tissue, adipose-derived stem cells, and aging. Plast Reconstr Surg.

[5] Delay E., Garson S., Tousson G., Sinna R. (2009). Fat injection to the breast: technique, results, and indications based on 880 procedures over 10 years. Aesthet Surg J.

[6] Shauly O., Gould D.J., Ghavami A. (2022). Fat grafting: basic science, techniques, and patient management. Plast Reconstr Surg Glob Open.

[7] Nemir S., Hanson S.E., Chu C.K. (2021). Surgical decision making in autologous fat grafting: an evidence-based review of techniques to maximize fat survival. Aesthet Surg J.

[8] Huang Y., Wu Q., Tam P.K.H. (2022). Immunomodulatory mechanisms of mesenchymal stem cells and their potential clinical applications. Int J Mol Sci.

[9] Xue E.Y., Narvaez L., Chu C.K., Hanson S.E. (2020). Fat processing Techniques. Semin Plast Surg..

[10] Zhang Z., Qiu L., Cui D., Geng J., Yi C. (2022). Use of platelet-rich fibrin in fat grafts during facial lipostructure. Front Surg.

[11] Bora P., Majumdar A.S. (2017). Adipose tissue-derived stromal vascular fraction in regenerative medicine: a brief review on biology and translation. Stem Cell Res Ther.

[12] Hong P., Yang H., Wu Y., Li K., Tang Z. (2019). The functions and clinical application potential of exosomes derived from adipose mesenchymal stem cells: a comprehensive review. Stem Cell Res Ther.

[13] Jeyaraman M., Muthu S., Sharma S., Ganta C., Ranjan R., Jha S.K. (2021). Nanofat: a therapeutic paradigm in regenerative medicine. World J Stem Cells.

[14] Copcu H.E., Oztan S. (2021). Not stromal vascular fraction (SVF) or nanofat, but total stromal-cells (TOST): a new definition. Systemic review of mechanical stromal-cell extraction techniques. Tissue Eng Regen Med.

[15] COLEMAN S. (1998). Structural fat grafting. Aesthet Surg J.

[16] Keck M., Zeyda M., Gollinger K. (2010). Local anesthetics have a major impact on viability of preadipocytes and their differentiation into adipocytes. Plast Reconstr Surg.

[17] Cucchiani R., Corrales L. (2016). The effects of fat harvesting and preparation, air exposure, obesity, and stem cell enrichment on adipocyte viability prior to graft transplantation. Aesthet Surg J.

[18] Mosmann T. (1983). Rapid colorimetric assay for cellular growth and survival: application to proliferation and cytotoxicity assays. J Immunol Methods.

[19] Gal S., Pu L.L.Q. (2020). An update on cryopreservation of adipose tissue. Plast Reconstr Surg.

[20] Smith P., Adams W.P., Lipschitz A.H. (2006). Autologous human fat grafting: effect of harvesting and preparation techniques on adipocyte graft survival. Plast Reconstr Surg.

[21] Troell R.J. (2025). Breast augmentation in body contouring using autologous stem cell-enriched fat grafting: fifteen-year clinical experience. J Clin Med.

[22] Karina K., Biben J.A., Ekaputri K. (2025). Revisiting fat graft harvesting and processing technique to optimize its regenerative potential. Plast Reconstr Surg Glob Open.

[23] Eskalen A., Işık E., Ozdemir I., Keskin I., Keskin M., Karacaoglan N. (2025). Evaluation of perilipin expression in centrifuged fat grafts on different revolutions per minute and duration combinations. Aesthetic Plast Surg.

[24] Francis A., Wang W.Z., Goldman J.J., Fang X.H., Williams S.J., Baynosa R.C. (2019). Enhancement of viable adipose-derived stem cells in lipoaspirate by buffering tumescent with sodium bicarbonate. Plast Reconstr Surg Glob Open.

[25] Bugajska-Liedtke M., Fatyga N., Brzozowski A., Bajek A., Maj M. (2024). Anaesthetics reduce the viability of adipose-derived stem cells. Adipocyte.

